# Potential Involvements of Cilia-Centrosomal Genes in Primary Congenital Glaucoma

**DOI:** 10.3390/ijms251810028

**Published:** 2024-09-18

**Authors:** Goutham Pyatla, Meha Kabra, Anil K. Mandal, Wei Zhang, Ashish Mishra, Samir Bera, Sonika Rathi, Satish Patnaik, Alice A. Anthony, Ritu Dixit, Seema Banerjee, Konegari Shekhar, Srinivas Marmamula, Inderjeet Kaur, Rohit C. Khanna, Subhabrata Chakrabarti

**Affiliations:** 1 Kallam Anji Reddy Molecular Genetics Laboratory, Prof. Brien Holden Eye Research Center, L.V. Prasad Eye Institute, Hyderabad 500034, Telangana, India; goutham@lvpei.org (G.P.); meha24k@gmail.com (M.K.); ashish.mishra@lvpei.org (A.M.); samir.bera@lvpei.org (S.B.); rathisonika7@gmail.com (S.R.); satishbiochem1@gmail.com (S.P.); aliceanthony94@gmail.com (A.A.A.); chavi.ritu3189@gmail.com (R.D.); inderjeet@lvpei.org (I.K.); 2 Manipal Academy of Higher Education, Manipal 576104, Karnataka, India; 3 Jasti V Ramanamma Children’s Eye Care Centre, L.V. Prasad Eye Institute, Hyderabad 500034, Telangana, India; mandal@lvpei.org; 4 Department of Ophthalmology, UMASS Medical School, Worcester, MA 01605, USA; wei.zhang4@astellas.com; 5 Gullapalli Pratibha Rao International Centre for Advancement of Rural Eye Care, L.V. Prasad Eye Institute, Hyderabad 500034, Telangana, India; seemadrishti@gmail.com (S.B.); kshekhar@lvpei.org (K.S.); sri.marmamula@lvpei.org (S.M.); rohit@lvpei.org (R.C.K.)

**Keywords:** primary congenital glaucoma, anterior segment, intraocular pressure, centrosome, cilia, gene, *CEP164*, *INPP5E*, *CYP1B1*

## Abstract

Primary congenital glaucoma (PCG) occurs in children due to developmental abnormalities in the trabecular meshwork and anterior chamber angle. Previous studies have implicated rare variants in *CYP1B1*, *LTBP2*, and *TEK* and their interactions with *MYOC*, *FOXC1*, and *PRSS56* in the genetic complexity and clinical heterogeneity of PCG. Given that some of the gene-encoded proteins are localized in the centrosomes (*MYOC*) and perform ciliary functions (*TEK*), we explored the involvement of a core centrosomal protein, *CEP164*, which is responsible for ocular development and regulation of intraocular pressure. Deep sequencing of *CEP164* in a PCG cohort devoid of homozygous mutations in candidate genes (n = 298) and controls (n = 1757) revealed *CEP164* rare pathogenic variants in 16 cases (5.36%). Co-occurrences of heterozygous alleles of *CEP164* with other genes were seen in four cases (1.34%), and a physical interaction was noted for CEP164 and CYP1B1 in HEK293 cells. Cases of co-harboring alleles of the *CEP164* and other genes had a poor prognosis compared with those with a single copy of the *CEP164* allele. We also screened *INPP5E*, which synergistically interacts with *CEP164*, and observed a lower frequency of pathogenic variants (0.67%). Our data suggest the potential involvements of *CEP164* and *INPP5E* and the yet unexplored cilia-centrosomal functions in PCG pathogenesis.

## 1. Introduction

Primary congenital glaucoma (PCG) is a rare autosomal recessive disease in children that occurs due to developmental defects in the trabecular meshwork (TM) and anterior chamber angle with a corresponding rise in intraocular pressure (IOP), optic nerve damage, and loss of vision [1,2]. PCG is clinically and genetically heterogeneous, and its molecular etiology is poorly understood [3]. Primarily, pathogenic variants in cytochrome P450 family 1 subfamily B member 1 (*CYP1B1*) [4,5,6], latent transforming growth factor beta binding protein 2 (*LTBP2*) [7,8], and *TEK* receptor tyrosine kinase (*TEK*) [9,10] have been implicated in PCG, but collectively they do not explain the entire molecular bases of this disease [3,11]. Additionally, variants in other genes in the anterior segment, cell signaling, extracellular matrix, and so on have been identified through GWAS (genome-wide association study) and deep sequencing approaches, but their specific role(s) in PCG pathogenesis remains elusive [3,12]. Variants in other genes comprising myocilin (*MYOC*) [13], forkhead box C1 (*FOXC1*) [14], angiopoietin 1 (*ANGPT1*) [15], optineurin (*OPTN*) [16], thrombospondin 1 (*THBS1*) [17], guanylate cyclase activator 1C (*GUCA1C*) [18], procollagen-lysine,2-oxoglutarate 5-dioxygenase 2 (*PLOD2*) [19], collagen type I alpha 1 chain (*COL1A1*) [20], forkhead box C2 (*FOXC2*), and paired like homeodomain 2 (*PITX2*) [21], neurotrophin 4 (*NTF4*) [22], C3, and PZP like alpha-2-macroglobulin domain containing 8 (*CPAMD8*) [23], sushi, von Willebrand factor type A, EGF, and pentraxin domain containing 1 (*SVEP1*) [24] and serine protease 56 (*PRSS56*) [25] are reported to be infrequent causes of PCG.

Centrosomal and ciliary genes play critical roles in various developmental anomalies in the anterior segment of the eye [26,27,28]. A juvenile open-angle glaucoma (JOAG)-associated gene, *MYOC,* localizes in the centrosome and the cytoplasmic filaments of TM cells and, to a lesser degree, in the trabecular beams and extracellular matrices in the juxta canalicular region of the TM [29,30]. It also co-localizes with cytochrome c oxidase subunit II of the mitochondria in the TM cells [29]. *MYOC* is also involved in PCG through its digenic interactions with *CYP1B1* [13].

The TM of the eye harbors primary cilia that help in regulating IOP by mechanosensation [31]. Elevated IOP causes the shortening of the cilia along with expressions of pro-inflammatory cytokines [31,32]. Thus, ciliary dysfunction could be an important contributor to the pathophysiology of glaucoma [33,34]. Earlier, we demonstrated the potential role of a ciliary gene *TEK* in PCG pathogenesis based on its genetic and physical interactions with *CYP1B1* [10]. *TEK* principally localizes to the primary cilia of the surface epithelium of the ovary, bursa, and extra-ovarian rete ducts and to the plasma membranes of ovarian theca and endothelial cells [35]. It has also been localized to the caveolae enriched with various signaling molecules [36] and in *ANGPT1* (Angiopoietin 1)-mediated cell–cell junctions in human umbilical vein endothelial cells (HUVECs) [9,10].

We explored the potential involvement of a centrosomal gene (*CEP164*), which is required for microtubule organization along with maintenance of the primary cilia and genomic stability [37,38] in PCG. Additionally, the *Cep164*^-/-^ mice have been found to have a lack of connection between cilia and outer segments of the photoreceptors [33]. *CEP164* interacts with *INPP5E* (Inositol Polyphosphate 5-Phosphatase E), which localizes in the cilia and helps in the stability and maintenance of the ciliary structures [39,40,41]. Dysfunction of *INPP5E* leads to shortened cilia and impaired ciliary function [42].

Mutations in *CEP164* and *INPP5E* have been implicated in retinal ciliopathies, and their functional interactions have already been deciphered [43,44,45,46,47]. Additionally, *CEP164* is involved in the regulation of epithelial to mesenchymal transition [48] and *INPP5E* in embryonic neural development [49]. Based on their potential roles in the centrosome and cilia, along with their implications in ocular development and regulation of IOP, we aimed to understand the involvement of *CEP164* and *INPP5E* in the PCG pathogenesis.

## 2. Results

### 2.1. Identification of Rare and Common Variants in CEP164 and INPP5E

**Figure 1:**
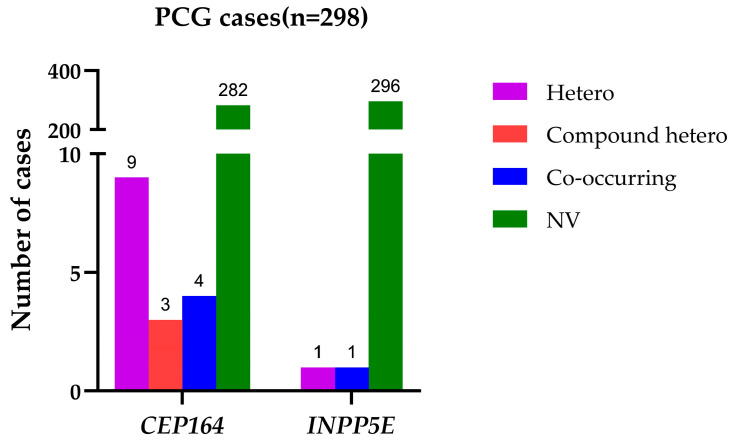
Distributions of rare variants in *CEP164* and *INPP5E* genes in PCG cases. Hetero = Cases with unique heterozygous alleles of either of these genes; Compound hetero = Cases with two heterozygous alleles within the *CEP164* and *INPP5E* gene, respectively; Co-occurring = Cases with co-occurring alleles of *CEP164* and *INPP5E*, and PCG-associated candidate genes, res; NV = Cases without any variations in the *CEP164* and *INPP5E* genes.

**Figure 2:**
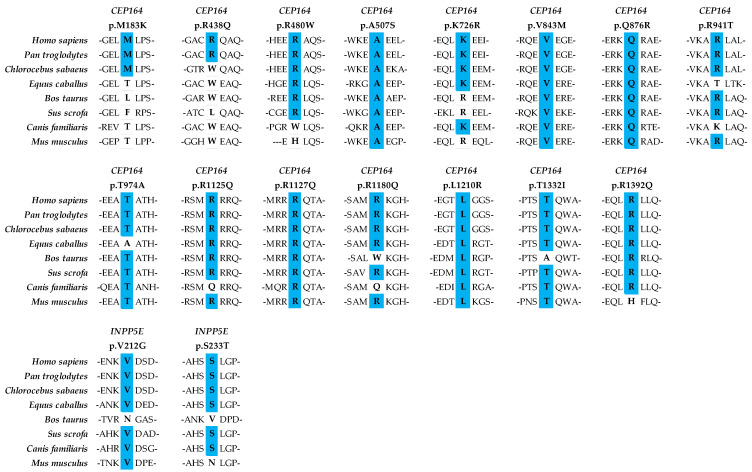
Conservation of wild-type residues of the rare variants across multiple species in CEP164 and INPP5E. Multiple sequence alignment was done using Clustal W and Clustal X v2.1 on Jalview v2.11 with UniProt database, and a schematic was prepared in Excel. CEP164 protein identifiers: *Homo sapiens* = Q9UPV0, *Pan troglodytes* = K7D7G7, *Chlorocebus sabaeus* = A0A0D9S3C3, *Equus caballus* = A0A3Q2I1M0, *Bos taurus* = F1MNI1, *Sus scrofa* = A0A8W4FCK4, *Canis familiaris* = A0A8I3MTC7, *Mus musculus* = Q5DU05. INPP5E protein identifiers: *Homo sapiens* = Q9NRR6, *Pan troglodytes* = K7B557, *Chlorocebus sabaeus* = A0A0D9RTF7, *Equus caballus* = A0A3Q2HCU7, *Bos taurus* = E1BAU6, *Sus scrofa* = A0A287AEE9, *Canis familiaris* = A0A8I3N0Y9, *Mus musculus* = Q9JII2.

We identified 17 heterozygous missense rare variants across these two genes in our PCG cohort (Table 1). There were relatively more PCG-associated variants in *CEP164* compared with the *INPP5E* gene. There were no homozygous pathogenic changes in either of these genes, and the distributions of overall variants comprised unique heterozygous alleles, compound heterozygous alleles (two heterozygous alleles within the same gene), and co-occurring alleles (co-occurrences of heterozygous alleles of *CEP164* and *INPP5E* along with PCG-associated candidate genes, such as *CYP1B1*, *LTBP2*, *TEK*, and *MYOC*) (Figure 1). The *INPP5E* variants exhibited relatively higher REVEL scores compared with the *CEP164* variants. All these rare variants were either absent or infrequently present among our ethnically matched control subjects and in the general populations of other global databases (Table 1). Most of these variants were highly conserved across multiple species (Figure 2).

We observed common variants in *CEP164* (n = 17) and *INPP5E* (n = 12) in our normal controls. The overall profile of allele frequencies of these common variants in our controls to other global databases is provided in Figure S1. Hardy–Weinberg equilibrium (HWE) analysis was performed for all the common variants of *CEP164* and *INPP5E* genes in normal controls, and distributions of their minor allele frequencies (MAFs) are provided in Table S1. Only polymorphic alleles with MAF > 0.05 and in HWE (*p* > 0.001) were included.

Genetic associations were performed by taking the common variants of *CEP164* (n = 10) and *INPP5E* (n = 9) genes, which were in HWE in our controls. Analysis of genetic associations of the common alleles between PCG cases and controls revealed that the “G” allele of rs73566945 variant (*INPP5E*) was significantly associated with the risk of PCG (*p* = 0.003; OR = 2.05; 95% CI, 1.27–3.29), which withstood statistical correction (corrected *p* value = 0.030) based on 10,000 permutations test (using Haploview). Genetic association for the rs73566945 genotypes was also undertaken. The homozygous genotype (GG) was significantly associated with PCG (*p* = 0.013; OR = 1.84, 95% CI, 1.13–2.99). The other common variants across these genes did not exhibit any association.

### 2.2. Haplotype Analysis of CEP164 and INPP5E Genes

The intragenic variants of *CEP164* and *INPP5E* were used to generate linkage disequilibrium (LD) plots (Figure S2), followed by haplotype analysis. There was tight LD across the markers for both genes. The haplotypes ‘C-C’ and ‘G-C’ (rs59763167-rs521099) and ‘A-A-A’ (rs33982662-rs34936112-rs73566945) of *CEP164* and *INPP5E*, respectively, were overrepresented either in the PCG cases or controls and exhibited a possible trend of association (based on uncorrected *p* value) but did not withstand statistical correction (Table 2). On the other hand, the ‘A-A-G’ (*INPP5E*) haplotype was significantly associated with the risk of PCG, which withstood statistical correction (based on 10,000 permutations test), which was largely contributed by the risk allele (G) of rs73566945. However, the rare variants across these two genes were not restricted to the risk haplotypes and were present in the background of other haplotypes as well.

### 2.3. Genetic and Physical Interactions between CEP164 and CYP1B1

We observed that the heterozygous rare variants of *CEP164* and *INPP5E* co-occurred with pathogenic variants of PCG-associated candidate genes (*CYP1B1*, *LTBP2*, *TEK*, and *MYOC*) in 5/298 (1.67%) cases (Figure 1). This indicated a possible multi allelic interactions of these genes in PCG pathogenesis. The distributions of the interacting alleles of these genes are provided in Supplementary Table S2.

**Figure 3:**
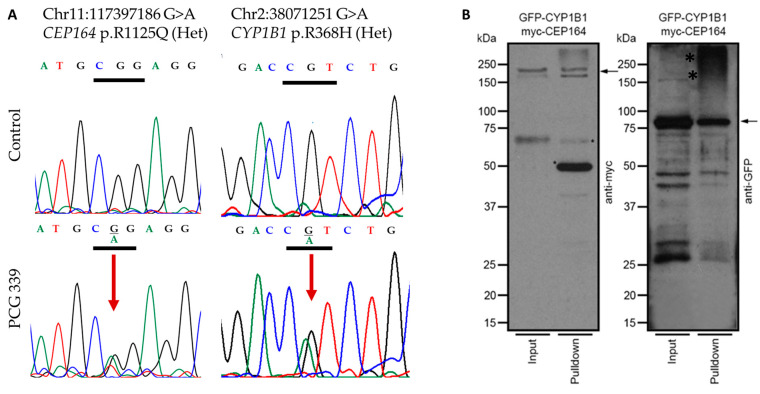
(**A**) Representative chromatograms indicating genetic interactions of *CEP164*: p.R1125Q and *CYP1B1*: p.R368H heterozygous rare variants in PCG 339 patient. The upper panel shows the wild-type sequence, and the lower panel indicates the variant marked by a red arrow. (**B**) HEK293 cells were transiently transfected with plasmids encoding GFP-CYP1B1 and MYC-CEP164. As a negative control, only GFP-encoding plasmid was utilized [10]. The cell extracts were subjected to immunoprecipitation (IP; pull-down) using anti-GFP or anti-myc antibodies, followed by SDS-PAGE and immunoblotting using anti-GFP or anti-myc antibodies, respectively. Arrows indicate the specific and expected protein bands (myc-CEP164 in the left panel and GFP-CYP1B1; kDa: kilo Daltons) detected by the indicated antibodies. Input lanes represent 20% of the protein used for IP. *: non-specific signal; Lower bands are likely the products of degradation. The negative control pull-downs were performed with protein extracts expressing the tags only, while the positive control was CYP1B1-TEK pull-down. These were previously published by our group [10].

We next assessed potential physical interaction between CYP1B1 with recombinant epitope-tagged versions of these proteins in HEK293 cells. We selected CEP164 for this experiment because of its known localization to centrosomes and a previously reported observation of myocilin, another known glaucoma-related gene in the centrosomes. The HEK293 cells co-transfected with plasmids encoding GFP-CYP1B1 and MYC-CEP164 showed that CEP164 interacts with CYP1B1 (Figure 3).

### 2.4. Genotype–Phenotype Correlation

Genotype–phenotype correlation was based on IOP, corneal diameter (CD), cup-to-disc ratios (CDRs), and visual acuity (VA) at presentation and follow-ups at 3 months and one year amongst PCG cases harboring rare variants in the *CEP164* gene. These cases were further classified based on their allelic configurations pertaining to the presence of either one heterozygous allele of a gene or combinations of multiple alleles based on co-occurring alleles of other candidate genes (Table 3). We could not perform a similar analysis for *INPP5E* due to a lack of PCG cases harboring rare variants in this gene (Table 1).

PCG cases harboring heterozygous alleles of *CEP164*, along with other candidate genes, had a relatively poor prognosis compared with those who harbored only a single copy of the *CEP164* allele. The mean IOP was uncontrolled and significantly raised in these cases at one-year follow-up (*p* = 0.019). Similarly, the cup-to-disc ratios were significantly higher in these cases at presentation (*p* = 0.004). However, visual acuity (VA) could not be graded at presentation or immediate follow-up in cases harboring *CEP164* variants (Table 3).

## 3. Discussion

PCG is a complex disease and is collectively attributed to multiple gene variants with varying magnitudes of effects. The PCG-associated candidate genes (*CYP1B1*, *LTBP2*, *TEK*, *MYOC*, and *FOXC1*) do not contribute to our complete understanding of the underlying molecular bases of this disease [3,10,11]. The discovery of novel genes and their functional interactions have provided new insights into biological pathways that may be implicated in PCG pathogenesis. Our earlier efforts in this direction have led to the identification of genic interactions of *CYP1B1* with *MYOC* [13], *FOXC1* [14], and *PRSS56* [25] in PCG. Additionally, we also demonstrated physical interactions of *CYP1B1* with *TEK* (a ciliary gene) due to their digenic involvement in PCG [10]. We have now extended our efforts toward understanding the possible involvements of centrosomal (*CEP164*) and its interacting ciliary (*INPP5E*) gene in PCG cases. Based on its genetic profile, functional interaction, and genotype–phenotype correlation, our study suggested that the *CEP164* gene could be a novel candidate in PCG pathogenesis.

We identified multiple novel rare variants in the highly conserved regions of *CEP164* and *INPP5E* in PCG cases devoid of homozygous mutations in the known PCG-associated candidate genes (Table 1; Figure 2). It is intriguing that we did not observe any homozygous pathogenic alleles in these genes, unlike in most other PCG-associated genes. The overall allelic contributions of *CEP164* (2.51%) and *INPP5E* (0.33%) in PCG were very low compared with the major candidate gene, *CYP1B1* (38.07%). However, their frequencies were comparable to other genes, comprising *LTBP2*, *TEK*, *MYOC*, and *FOXC1*, which ranged from 0.31 to 3.55% in our PCG cohort.

**Figure 4:**
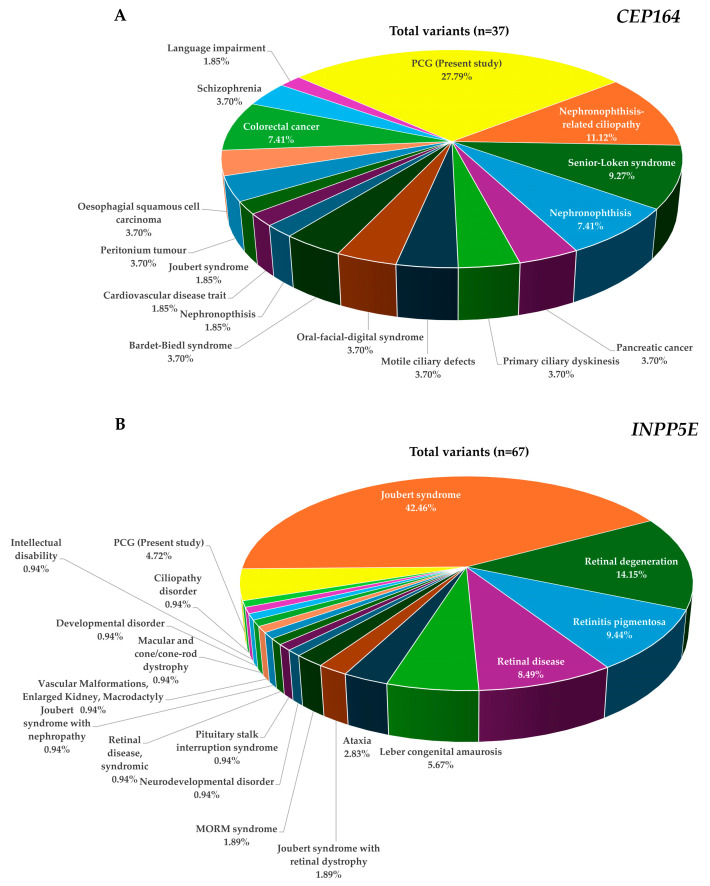
Pie charts depicting the proportions of observed pathogenic variants in *CEP164* (**A**) and *INPP5E* (**B**) across different phenotypes and the present study (PCG). The data show the frequencies of the classified disease mutations as per the HGMD database (Tables S3 and S4).

CEP164 is involved in microtubule organization and maintenance for the formation of primary cilia, which are essential for the proper functioning of the TM and the anterior chamber angle [37,38]. Additionally, it is also involved in DNA damage response and chromosome segregation, which are critical processes in maintaining genomic stability. Mutations in *CEP164* have been associated with severe ciliopathy phenotypes (Figure 4A), such as nephronophthisis, occipital encephalocele, and liver fibrosis, and milder phenotypes, like nephronophthisis with Leber congenital amaurosis [33,44,45,50,51]. The exact mechanisms by which *CEP164* would contribute to PCG are not fully understood, but mutations in this gene may possibly interfere with cell cycle progression, apoptosis, and epithelial-to-mesenchymal transition during developmental stages [48].

We also observed co-occurrences of heterozygous pathogenic alleles of *CEP164* (n = 4) that genetically interact with heterozygous alleles of other PCG-associated genes (Figure 1). Interestingly, the genetic interaction of *CEP164* (p.R1125Q) with *CYP1B1* (p.R368H) was supported by a corresponding physical interaction in HEK293 cells (Figure 3), like our earlier observations on *TEK* and *CYP1B1* [10]. We speculate that co-occurring mutations in *CEP164* and *CYP1B1* (Figure 3) may perturb this interaction and disrupt ciliary functions and regulation of IOP. However, this needs to be confirmed with additional functional validations.

We have also demonstrated that another JOAG-associated gene, *MYOC*, localizes in the centrosome and genetically interacts with *CYP1B1* through a digenic mechanism in PCG [13]. While we did not observe any genetic interactions of *MYOC* and *CEP164* alleles in our present cohort, the presence of pathogenic variants of *MYOC* in PCG in our earlier study [13] and in other populations [22,52,53,54,55,56,57,58,59] suggest the potential involvement of centrosomal proteins in PCG, which need to be functionally characterized.

Interestingly, PCG patients harboring heterozygous alleles of *CEP164*, along with heterozygous alleles of other PCG-associated genes had a relatively poor prognosis in terms of their IOP control and cup-to-disc ratios (Table 3). This has been consistently observed with other multi-allelic scenarios involving *MYOC* [13] and *TEK* [10] with *CYP1B1* in our PCG cohort.

The *INPP5E*, on the other hand, is a widely expressed ciliary gene that plays a critical role in controlling ciliary function by regulating the length of the cilia [42]. Mutations in *INPP5E* have been associated with various ciliopathies, including Joubert syndrome, which can present with glaucoma as one of the clinical features (Figure 4B). Our data revealed relatively smaller numbers of rare variants in *INPP5E* compared with *CEP164* (Table 1). There was only one co-occurring allele of *INPP5E* with another gene (*MYOC*), and genotype–phenotype correlation was inconclusive considering the number of PCG patients harboring rare variants in this gene (Table 3). Although there was no evidence of any genetic interactions between *CEP164* and *INPP5E* genes, it could be speculated that defects in *INPP5E* may lead to shortened cilia and impaired ciliary function, which may further contribute to the development of PCG [40,42].

Overall, *CEP164* and *INPP5E* have revealed a total of 22 and 65 pathogenic variants, respectively, across multiple phenotypes as per the HGMD (Human Gene Mutation database) [60]. The discovery of rare variants in these genes in PCG has expanded the mutation spectra of *CEP164* (n = 37) and *INPP5E* (n = 67) genes (Figure 4A,B; Tables S3 and S4). We observed a relatively larger number of rare variants in *CEP164* compared with *INPP5E* in PCG (Figure 4). The observation of a functional interaction and poor prognosis in cases with co-occurring alleles of *CEP164* and other genes, along with a strong pathogenic potential of *INPP5E,* might be indicative of their underlying role(s) in PCG that need to be elucidated further in additional cohorts.

**Figure 5:**
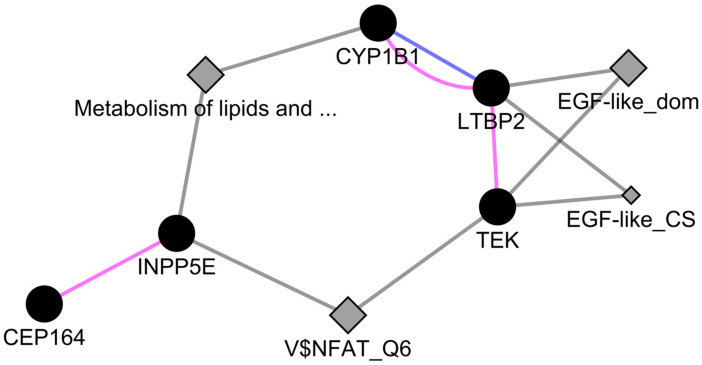
Network analysis of *CEP164* and *INPP5E* with PCG-associated candidate genes. The network was generated using GeneMania of Cytoscape v3.5.3 software. Solid nodes represent genes. Diamond nodes represent the consolidated pathways, domains and transcription factor targets. Color scheme: Pink = Co-expression, Blue = Co-localization, Grey = Shared attribute. “V$NFAT_Q6” = Transcription factor target motif, “EGF-like_dom” = EGF-like domain, “EGF-like_CS” = EGF-like conserved site, “Metabolism of lipids and …” = Metabolism of lipids and lipoproteins.

Network analysis of *CEP164* and *INPP5E*, along with PCG-associated candidate genes, indicated their interactions through pathways, which is further suggestive of their involvement with PCG (Figure 5). Further, metabolic interactions of lipid and lipo-proteins were noted between *CYP1B1* and *INPP5E* (that further interacts with *CEP164*). On the other hand, *TEK* and *INPP5E* shared a common transcription factor target. The involvement of these genes in ciliopathies and various retinal functions indicated a possible mechanism in PCG pathogenesis [44,45,46,47].

*CEP164* is also involved in retinal photoreceptor layer development [33] and *INPP5E* in embryonic neural development [49]. Both these genes synergistically interact with each other in the formation of a functional network involving the primary cilia [43]. Based on the involvement of these genes in our cohort, we speculate their involvement in retinal damage in PCG. Reduction in retinal nerve fiber layer thickness (RNFL) and its correlation with IOP among PCG patients [61,62] have already indicated the involvement of retinal-associated genes in disease pathogenesis. This is further supported by the fact that the primary PCG-associated gene, *CYP1B1,* is expressed in retinal ganglion cells (RGCs) and promotes its survival [63], but the possible mechanisms leading to retinal damage in PCG remains elusive.

In summary, *CEP164* and *INPP5E* genes may play critical roles in PCG considering their involvements in centrosomal, ciliary, and retinal functions and the regulation of IOP. Further research would be needed to fully understand their underlying molecular mechanisms through which these genes contribute to PCG pathogenesis.

## 4. Materials and Methods

### 4.1. Study Approval

The study was approved by the Institutional Review Board of L V Prasad Eye Institute (LEC 09-18-141) and adhered to the tenets of the Declaration of Helsinki. A written informed consent was obtained from all the study subjects and guardians of minors.

### 4.2. Enrolment of Cases and Controls

The study comprised 2055 subjects, including PCG cases (n = 298) and ethnically matched normal controls (n = 1757). Detailed inclusion and exclusion criteria have been described earlier [10,64]. Briefly, all study participants underwent a comprehensive ocular examination. Each case was independently diagnosed by at least two glaucoma specialists, and a good inter-observer agreement was seen based on kappa statistics (κ = 0.94). Cases that had discordant diagnoses amongst the clinicians were excluded. Demographic details of subjects, including their gender, history of consanguinity, and age at disease onset and intervention, were recorded. Quantitative data of clinical variables such as intraocular pressure (IOP), corneal diameter (CD), cup-to-disc ratio (CDR), and visual acuity (VA) were collected from all the PCG cases at presentation and further follow-ups following surgery.

### 4.3. Targeted Sequencing, Cell Culture, and Pull-Down Assay

A customized, targeted gene panel (Applied Biosystems, Foster City, CA, USA) comprising *CEP164* and *INPP5E* genes, along with other PCG-associated genes, was used for screening. Library preparation, amplification, enrichments, and deep sequencings were performed as per the manufacturer’s guidelines (Applied Biosystems, Foster City, CA, USA). The quality control measures for data cleaning, data analysis pipelines, and interpretations have been described earlier [10]. The observed variants were validated through Sanger sequencing using the BigDye Terminator (v3.1) chemistry on a 3130xl Genetic Analyzer (Applied Biosystems, Foster City, CA, USA). The cell culture experiments with HEK293 cells and GFP pull-down assay were as described earlier [10].

### 4.4. Allele and Haplotype Analysis

The Ensembl canonical transcripts of *CEP164*: ENST00000278935.8 and *INPP5E*: ENST00000371712.4 were considered for analysis. Allele frequencies were calculated using the gene counting method along with their odds ratios and 95% confidence intervals. *p* values were based on the Chi-square test. These frequencies were compared with the global allele frequencies reported in the 1000 Genomes [65], gnomAD [66], and All of Us [67] databases.

Pathogenicity predictions were based on REVEL scores [68], which provide a score combining 13 different tools (SIFT, PolyPhen2, Mutation Taster, Mutation Assessor, FATHMM v2.3, MutPred, VEST 3.0, PROVEAN, LRT, phyloP, SiPhy, GERP++, and phastCons).

Hardy–Weinberg equilibrium (HWE) and haplotypes analysis were conducted using the HaploView (version 4.2) software [69]. The HWE cut-off *p* value was 0.001 based on the default parameter of this software. The minimum genotype cut-off percent was set at 100%, and the minimum minor allele frequency (MAF) was >0.05. Only haplotypes with frequencies above 5% were considered. Phenotypes of genes and disease-causing mutations were extracted from the HGMD database (accessed on 19 July 2024) [60].

### 4.5. Conservation of Amino Acids

Multiple sequence alignment was performed using Clustal W and Clustal X v2.1 [70] on the Jalview v2.11 platform [71]. With three residues on either side for the target residue, the remaining intermediary amino acids were removed from the sequence.

### 4.6. Network Analysis

Network analysis was conducted using the GeneMania v3.5.3 app on the Cytoscape software platform [72]. Gene list as an input, co-expression, co-localization, genetic interactions, pathways, physical interactions, predicted interactions along with attributes including consolidated pathways, drug interactions, InterPro, miRNA-target-predictions, and transcriptional factor targets were analyzed. The top twenty related genes and, at most, twenty attributes were used with automatic weighting.

## 5. Conclusions

The potential functional involvement and genotype–phenotype correlation exhibited by *CEP164* variants, along with a strong pathogenic potential of *INPP5E* variant, suggest these genes as a potential candidate(s) in PCG and a yet unexplored involvement of cilia-centrosomal functions in disease pathogenesis.

## Figures and Tables

**Table 1:** A. Distributions of allele frequencies of rare variants in the *CEP164* gene in PCG. B. Distributions of rare variants in the *INPP5E* gene in PCG.

Chromosomal Position (GRCh38)	Amino Acid Change	dbSNP ID	REVEL Score	Cases MAF (n = 298)	Controls MAF (n = 1757)	*p* Value	Odds Ratio (95% CI)	1000 Genomes (n = 2504); gnomAD v4.1 (n = 807,162); All of Us (n = 245,400)
**A**								
11: 117361989T > A	p.M183K	rs144206271	0.18	0.0016	0.0003	0.15	5.91 (0.37–94.74)	0.0008; 0.00063; 0.0006
11: 117375787G > A	p.R438Q	rs137987733	0.05	0.0016	0.0003	0.15	5.91 (0.37–94.74)	NA; 0.00018; 0.0001
11: 117381729C > T	p.R480W	rs112209873	0.04	0.0016	0	-	-	0.001; 0.00019; 0.00068
11: 117381810G > T	p.A507S	NA	0.08	0.0016	0	-	-	NA
11: 117391109A > G	p.K726R	rs2044597036	0.02	0.0016	0	-	-	NA
11: 117393037G > A	p.V843M	rs566117718	0.09	0.0016	0.0008	0.55	1.96 (0.20–18.98)	0.0012; 0.00005; 0.000016
11: 117394360A > G	p.Q876R	rs752659513	0.13	0.0033	0.0003	0.01	11.86 (1.07–131.20)	NA; 0.000044; 0.000006
11: 117394981G > C	p.R941T	rs749310077	0.03	0.0016	0	-	-	NA; 0.0000006; NA
11: 117395553A > G	p.T974A	rs56699807	0.04	0.0033	0.0003	0.01	11.86 (1.07–131.20)	0.0002; 0.0003; 0.0003
11: 117397186G > A	p.R1125Q	rs767918200	0.03	0.0016	0.0008	0.55	1.96 (0.20–18.98)	NA; 0.000015; 0.000002
11: 117397192G > A	p.R1127Q	rs753895198	0.16	0.0016	0	-	-	NA; 0.000009; 0.000016
11: 117407962G > A	p.R1180Q	rs568896676	0.02	0.0050	0.0006	0.004	8.92 (1.48–53.61)	NA; 0.00001; 0.000012
11: 117408909T > G	p.L1210R	rs767571570	0.17	0.0016	0	-	-	NA; 0.000003; NA
11: 117409864C > T	p.T1332I	rs760788324	0.03	0.0016	0	-	-	NA; 0.000006; NA
11: 117411806G > A	p.R1392Q	rs772989312	0.07	0.0016	0.0011	0.72	1.47 (0.16–13.24)	NA; 0.000012; 0.000016
**B**								
9: 136438722C > G	p.S233T	rs568767788	0.35	0.0016	0.0003	0.15	5.91 (0.37–94.74)	0.00039; 0.000026; 0.000006
9: 136438785A > C	p.V212G	rs533861933	0.54	0.0016	0.0017	0.98	0.98 (0.11–8.18)	0.00019; 0.0000068; 0.00001

MAF = Minor allele frequency; NA = Not available.; - = Cannot be determined.

**Table 2:** Distributions of haplotypes in *CEP164* and *INPP5E* genes.

Genes	Haplotypes	Overall Haplotype Frequency	Frequency in Cases	Frequency in Controls	Chi Square	Uncorrected *p* Value	Corrected *p* Value *
*CEP164*	C-C	0.747	0.792	0.739	7.464	**0.006**	0.056
*CEP164*	C-G	0.198	0.171	0.203	3.179	0.075	0.414
*CEP164*	G-C	0.055	0.037	0.058	4.383	**0.036**	0.242
*INPP5E*	C-G-G	0.844	0.834	0.846	0.613	0.434	0.954
*INPP5E*	A-A-G	0.084	0.131	0.076	20.037	**7.6 × 10^−6^**	**0.0005**
*INPP5E*	A-A-A	0.055	0.032	0.059	7.234	**0.007**	0.055

Order of haplotypes for *CEP164* (rs59763167-rs521099) and *INPP5E* (rs33982662-rs34936112-rs73566945); * Based on 10,000 permutation test (Haploview v4.2 software). Bold format represents the significant *p* values.

**Table 3:** Genotype–phenotype correlation based on the presenting and follow-up IOP, corneal diameter, cup-to-disc ratio, and visual acuity in patients harboring various combinations of *CEP164* variants.

Genotype Combinations in PCG Cases (Number of Cases)	Intraocular Pressure (mmHg)			Cup-to-Disc Ratio			Corneal Diameter (mm)			Visual Acuity (logMAR)		
	At Presen-tation	After 3 Months	After 1 Year	At Presen-tation	After 3 Months	After 1 Year	At Presen-tation	After 3 Months	After 1 Year	At Presen-tation	After 3 Months	After 1 Year
Cases with unique heterozygous *CEP164* alleles only (n = 9)	25.75 ± 6.63	12.86 ± 3.8	11.63 ± 1.69	0.48 ± 0.13	0.54 ± 0.22	0.46 ± 0.2	13.06 ± 0.98	12.71 ± 1.19	13.1 ± 0.74	NA	NA	1.19 ± 0.47
Cases with compound heterozygous *CEP164* alleles (n = 3)	28 ± 6.93	15.33 ± 5.03	10.33 ± 2.52	NA	0.47 ± 0.23	0.5 ± 0.26	13.67 ± 0.58	13.33 ± 0.58	13.67 ± 0.29	NA	NA	
Cases with co-occurring *CEP164* alleles along with heterozygous alleles of other genes (n = 4)	24.25 ± 6.65	NA	17.67 ± 5.86	0.83 ± 0.06	NA	0.63 ± 0.31	15 ± 2.29	NA	NA	NA	NA	2.34 ± 0.75
* *p* value (Unique heterozygous *CEP164* alleles versus co-occurring *CEP164* and alleles of other genes)	0.72	NA	**0.019**	**0.004**	NA	0.284	0.056	NA	NA	NA	NA	0.053
* *p* value (Compound heterozygous *CEP164* alleles versus co-occurring *CEP164* and alleles of other genes)	0.5	NA	0.117	NA	NA	0.598	0.384	NA	NA	NA	NA	NA

* *p* value is based on Student’s *t* test; NA: Data are not available. Bold format represents the significant *p* values.

## Data Availability

All data are provided in the text and Supplementary Materials of this manuscript.

## Supplementary Materials

The following supporting information can be downloaded at https://www.mdpi.com/article/10.3390/ijms251810028/s1.
