# Supplementary material for: Potential Involvements of Cilia-Centrosomal Genes in Primary Congenital Glaucoma
Source: Int J Mol Sci. 2024 Sep 18;25(18):10028. doi: 10.3390/ijms251810028 (PMC11431959; doi:10.3390/ijms251810028)
Supplement: Supplementary file 1 [file ijms-25-10028-s001.zip › Figure S2_LD plots.pdf]

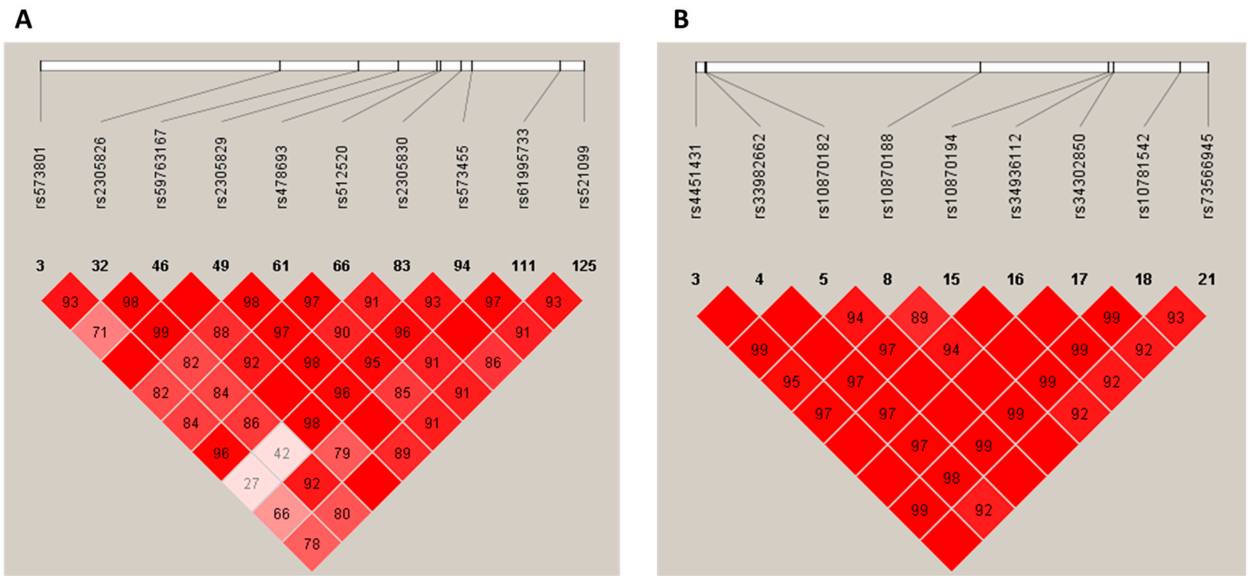

Figure S2: Linkage disequilibrium plots of *CEP164* (A), *INPP5E* (B). Upper horizontal white bar represents the chromosome and the corresponding positions of the SNPs. Colour scheme: Bright red: D′=1 and LOD >=2, Shades of pink/ red: D′ = <1 and LOD >=2, Blue: D′ =1 and LOD <2, White: D′ <1 and LOD <2. Values inside blocks represents the D′ value of the block. Empty red block indicates D′ =1.
