# Supplementary figures and images for: Potential Involvements of Cilia-Centrosomal Genes in Primary Congenital Glaucoma
Source: Int J Mol Sci. 2024 Sep 18;25(18):10028. doi: 10.3390/ijms251810028 (PMC11431959; doi:10.3390/ijms251810028)

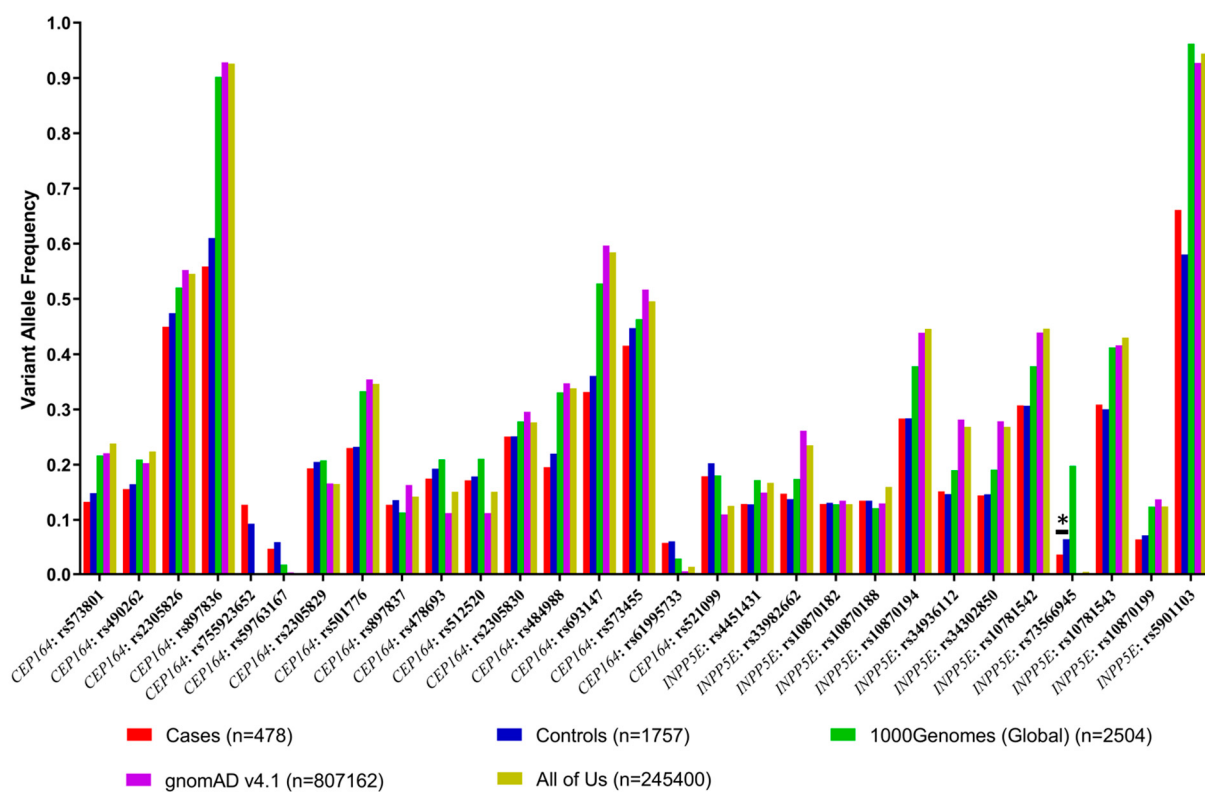

**Figure S1:** Distributions of allele frequencies of common variants in *CEP164* and *INPP5E*.

\* = P-Value < 0.05.

Supplement: Supplementary file 1 [file ijms-25-10028-s001.zip › Figure S1_Common Variants.pdf]
